# Testicular expression of *TDRD1*, *TDRD5*, *TDRD9* and *TDRD12* in azoospermia

**DOI:** 10.1186/s12881-020-0970-0

**Published:** 2020-02-14

**Authors:** Emad Babakhanzadeh, Ali Khodadadian, Saadi Rostami, Iraj Alipourfard, Mohsen Aghaei, Majid Nazari, Mehdi Hosseinnia, Mohammad Yahya Vahidi Mehrjardi, Yalda Jamshidi, Nasrin Ghasemi

**Affiliations:** 1 Department of Medical Genetics, Shahid Sadoughi University of Medical Sciences, Yazd, Iran; 2 Medical Genetics Research Center, Shahid Sadoughi University of Medical Sciences, Yazd, Iran; 3 Department of Cellular and Molecular Biology, Faculty of Science, Azarbaijan Shahid Madani University, Tabriz, Iran; 4 Center of Pharmaceutical Sciences, Faculty of Life Sciences, University of Vienna, Vienna, Austria; 5 School of Pharmacy, Faculty of Sciences, University of Rome Tor Vergata, Rome, Italy; 6 Department of Biology, Faculty of Science, University of Guilan, Rasht, Iran; 7 Genetics Centre, Molecular and Clinical Sciences Institute, St George’s University of London, London, UK; 8 Abortion Research Centre, Yazd Reproductive Sicences Institue, Shahid sadoughi University of Medical Sciences, Yazd, Iran

**Keywords:** Spermatogenesis, Non-obstructive Azoospermia, piRNAs, TDRD genes

## Abstract

**Background:**

Tudor domain-containing proteins (TDRDs) play a critical role in piRNA biogenesis and germ cell development. piRNAs, small regulatory RNAs, act by silencing of transposons during germline development and it has recently been shown in animal model studies that defects in *TDRD* genes can lead to sterility in males.

**Methods:**

Here we evaluate gene and protein expression levels of four key TDRDs (TDRD1*,* TDRD5*,* TDRD9 and TDRD12) in testicular biopsy samples obtained from men with obstructive azoospermia (OA, *n* = 29), as controls, and various types of non-obstructive azoospermia containing hypospermatogenesis (HP, 28), maturation arrest (MA, *n* = 30), and Sertoli cell-only syndrome (SCOS, *n* = 32) as cases. One-way ANOVA test followed by Dunnett’s multiple comparison post-test was used to determine inter-group differences in *TDRD* gene expression among cases and controls.

**Results:**

The results showed very low expression of *TDRD* genes in SCOS specimens. Also, the expression of *TDRD1* and *TDRD9* genes were lower in MA samples compared to OA samples. The expression of *TDRD5* significantly reduced in SCOS, MA and HP specimens than the OA specimens. Indeed, *TDRD12* exhibited a very low expression in HP specimens in comparison to OA specimens. All these results were confirmed by Western blot technique.

**Conclusion:**

TDRDs could be very important in male infertility, which should be express in certain stages of spermatogenesis.

## Background

Infertility remains a significant global burden [1]. Estimates suggest that 10–15% of couples worldwide experience infertility [2], with male infertility being the underlying cause in 20–50% of cases [3]. For the majority of cases however the etiology remains unknown, and is termed idiopathic infertility [4]. Azoospermia, absence of spermatozoa in the semen, is one of the most common reasons for infertility in men, with a prevalence of 1% in the general population, and over 15% in infertile men [5, 6]. Forty percent of cases are due to male genital system blockage (obstructive azoospermia) and in the remaining cases due to low sperm production (non-obstructive azoospermia - NOA) [7, 8].

PIWI-interacting RNA (piRNA) proteins have recently been shown to play an essential role in male fertility and spermatogenesis in humans [9]. The piRNA pathway-associated genes are highly expressed in germline cells [10]. piRNAs act by suppressing transposons and preventing their mobilization through both post-transcriptional and transcriptional mechanisms, such as degradation, chromatin remodeling, and DNA methylation [11–14].

P-element–induced wimpy testis (PIWI) proteins specifically recognize piRNAs to mediate transposon silencing. The Tudor domain of Tudor domain-containing proteins (TDRDs) have been shown to directly bind to PIWI to help regulate this process via their dimethylated arginine (sDMA) or lysine residues [15], potentially acting as a mediator or cofactor for protein-protein interactions in the piRNA pathway [16]. Twelve TDRDs have been identified in human and animal model studies, and defects in each of these can interfere with spermatogenesis [16–18].

Given the current knowledge that piRNAs and PIWI genes are involved in spermatogenesis, and the potential role of TDRDs in this process, we sought to evaluate TDRD gene expression in testicular tissue of patients with azoospermia.

## Methods

### Study participants

One hundred and nineteen NOA and OA men admitted to the Abortion Research Centre, Yazd Reproductive Sciences Institute, were entered into this study. All subjects were undergoing bilateral testicular tissue micro-dissection (mTESE) operations to attain spermatozoa for intracytoplasmic sperm injection (ICSI). The study was approved by the local Ethics Committee and written informed consent was obtained from all subjects. Preoperative tests included karyotyping and Y chromosome microdeletion analysis, and the levels of serum follicle stimulating hormone (FSH), luteinizing hormone (LH) and testosterone. Patients were not receiving hormone therapy and all had primary infertility. No history of TESE and cryptorchidism were reported for any of the participants. Patients with cystic fibrosis, chromosomal abnormalities and Y chromosome microdeletion were omitted from the study. Subjects with normal spermatogenesis were included as the control group. The best control samples were men with proven fertility, and the problem was that they never referred to infertility centers for mTESE surgery, so we used samples with normal spermatogenesis as the control group.

### Tissue acquisition and histological evaluation

Following TESE, testicular samples were split into three; one was fixed in Bouin’s solution for histological examination and two other sections instantly frozen in liquid nitrogen for RNA extraction and Western blot assay. Histological examination was carried out with hematoxylin and eosin (H&E) and interpreted by a trained pathologist to classify the samples with normal spermatogenesis (OA, *n* = 29), lack of germ cells (SCOS, *n* = 32), declined number of spermatozoa (HP, *n* = 28), and incomplete maturation of germ cells (MA, *n* = 30). No significant differences in age, LH, FSH, and testosterone serum concentrations were found between the OA, HP, MA, and SCOS groups (Table 1).

**Table 1:** The clinical features of patient groups

Patient groups	Number of patients	Genetic analysis	Age of patients (years)	LH (mIU/ml)	FSH (mIU/ml)	Testosterone (ng/ml)
OA	29	46XY/ normal AZF	34.3 ± 2.3	8.34 ± 1.12	9.21 ± 1.27	4.41 ± 0.34
HP	28	46XY/ normal AZF	31.5 ± 2.4	7.92 ± 1.18	9.32 ± 1.13	4.35 ± 0.22
MA	30	46XY/ normal AZF	33. 4 ± 2.1	7.93 ± 1.35	9.42 ± 1.61	4.43 ± 0.33
SCOS	32	46XY/ normal AZF	30.4 ± 2.1	8.62 ± 1.61	9.73 ± 1.42	4.45 ± 0.34
P-value	–	–	NS	NS	NS	NS

Values are mean ± standard deviation

*NS* Non-significant, *OA* Obstructive azoospermia, *HP* Hypospermatogenesis, *MA* Meiotic arrest, *SCOS* Sertoli cell only syndrome

### RNA extraction and cDNA synthesis

Frozen testis tissue was homogenized, and total RNA extracted using the RNeasy Plus Universal Mini Kit (Qiagen, Hilden, Germany) based on the manufacturer’s protocol and then stored at − 80 °C. In-solution DNase digestion was accomplished to eliminate DNA contamination. The concentration and purification of RNA was specified using Nanodrop2000 spectrophotometer (Thermo Scientific, Wilmington, USA) and confirmed by agarose gel electrophoresis. Template cDNA was synthesized from 1 μg of whole extracted RNA with Revert Aid First Strand cDNA Synthesis Kit (Thermo Scientific, Vilnius, Lithuania) using oligo dT and random hexamer primers simultaneously for each reaction in an Eppendorf Mastercycler Gradient (Hamburg, Germany).

### Quantitative RT-PCR

Initial denaturation for RT-PCR started at 95 °C for 8 min, followed by 40 cycles of denaturation at 95 °C for 10 s, annealing at 60 °C for 30 s, extension at 72 °C for 30 s (Table 1). The melting curve was formed by increasing the temperature from 72 to 95 °C to omit genomic DNA or amplification of primer dimers. qPCR was in triplicates on 48-well plates Step-One-Plus RT-PCR System (Applied Biosystems) by 1.0 μl of produced cDNA, 10 μl of the SYBR Green master mix (Applied Biosystems ABI/PE, Foster City, CA), and 7.0 μl of DNase/RNase-free water, 1 μl of designing primers (Table 2) for the gene expression profile. The average CT was used for further analysis, and all RT–PCR runs contained non template (cDNA) controls in order to reject potential contamination. Relative gene expression analysis was performed according to the comparative CT method: 2^-∆∆CT^ for TDRD1, TDRD5, TDRD9 and TDRD12. The 2^-∆∆CT^ parameter displays the expression fold of *TDRD* with respect to the housekeeping *ACTB* gene.

**Table 2:** qPCR primers used in this study

Gene	Primers (5′ → 3′)	Product size (bp)	TM (°C)
*TDRD1*	F: TCCTCTTCGGTCCACAACTT	197	59
	R: CCTCCACATCCTTTGTTTCAA		60
*TDRD5*	F: AAGTTCCCAGAGGGTTTGTTT	194	58
	R: AGAGGCTTCTTATCCGCAT		59
*TDRD9*	F: GCCAGGTCTGGGTGAAATAA	171	58
	R:TCTGCAATATTGGTGGACAGA		58
*TDRD12*	F: TCGTTTATGCAGCTTCCCTA	172	57
	R: CCACCTGGGTAGTTGCTTT		58
*ACTB*	F: AGCACAGAGCCTCGCCTT	172	60
	R: AGGGCATACCCCTCGTAGAT		60

*bP* Base pairs, *F* Forward, *R* Reverse, *TM* Melting temperature

### Western blot

Western blot examination was accomplished as formerly explained [19]. Briefly, equal amounts of proteins (35 mg) obtained from testis samples were separated with 12% SDS-PAGE and electrotransferred onto nitrocellulose paper. The membranes were blocked with (2–5%) non-fat dry milk in 1x TBST (10 mM/L Tris-HCl, pH 8.0; 150 mM/L NaCl, 0.1% Tween 20) for 1 h at 25–30 °C, and then incubated with primary TDRD1(1:300; No. ABIN2373082; Antibodies-online, Aachen, Germany), TDRD5 (1:300; No. ABIN4358317; Antibodies-online, Aachen, Germany), TDRD9 (1:300; No. ABIN2373082; Antibodies-online, Aachen, Germany), TDRD12 (1:300; No. ABIN6769800; Antibodies-online, Aachen, Germany), ACTB (1:300; No. ABIN4284408; Antibodies-online, Aachen, Germany) antibody at 4 °C overnight, then with secondary goat anti-human-IgG-(H-L) antibody conjugated with horseradish peroxidase (HRP) (1:500; No. SA00001–17; ProteinTech, Manchester, M3 3WF United Kingdom) for 1 h at 25 °C. At the end, the immunoreactive signals were determined via the ECL kit (Thermo Scientific).

### Statistical analysis

The results are verified as the mean ± SEM. Statistical analysis was done using one way anova test followed by Dunnett’s multiple comparison post-test. *P*-values less than 0.05 were deemed to be statistically significant. Statistical analysis was implemented via GraphPad Prism 6 software.

## Results

**Fig. 1:**
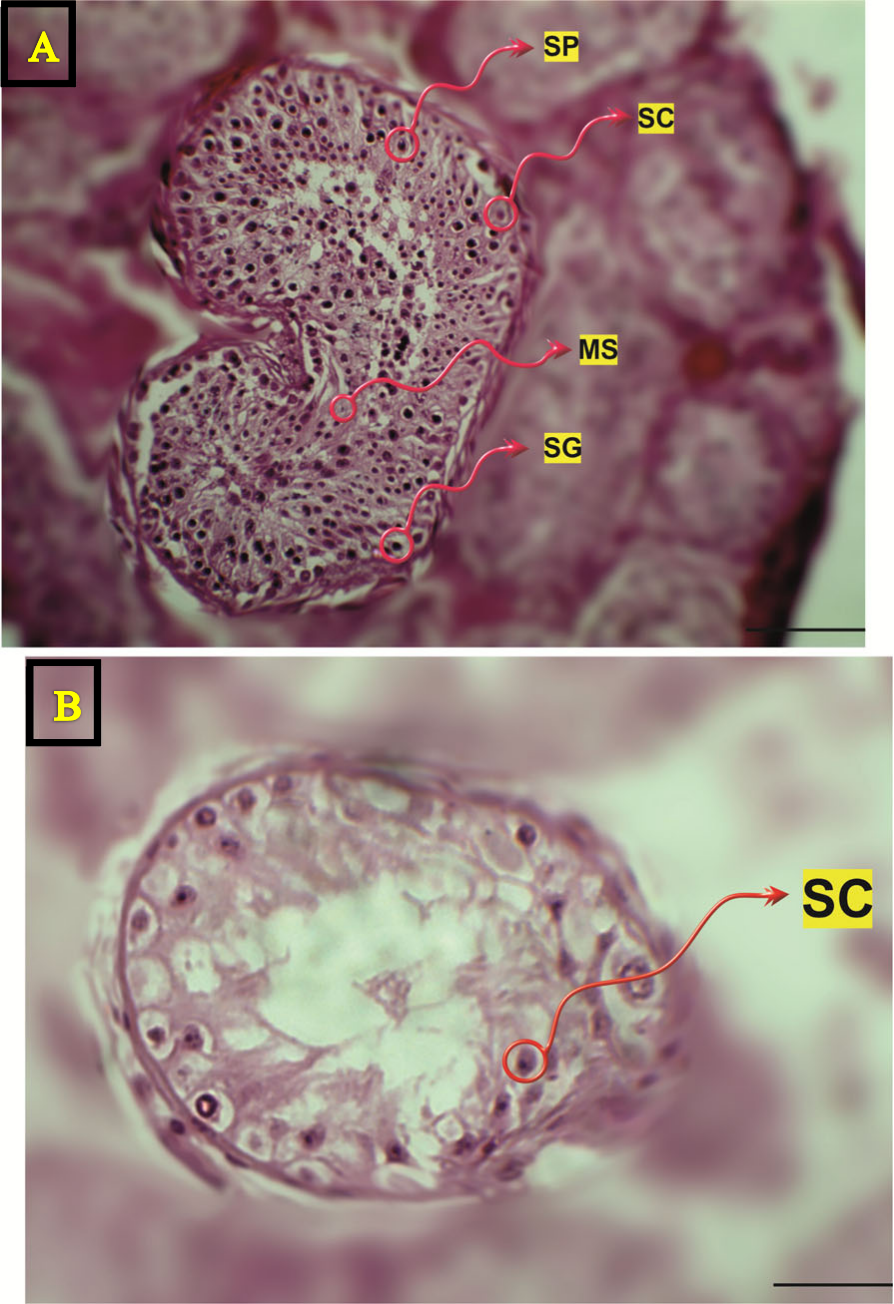
**a** Testicular sections of samples with OA (obstructive azoospermia). **b** Testicular segments of specimens by SCOS (Sertoli cell-only syndrome). SP = spermatocyte; MS = mature spermatid; SC = Sertoli cell; SG = spermatogonia; Scale bar = 20 μm

**Fig. 2:**
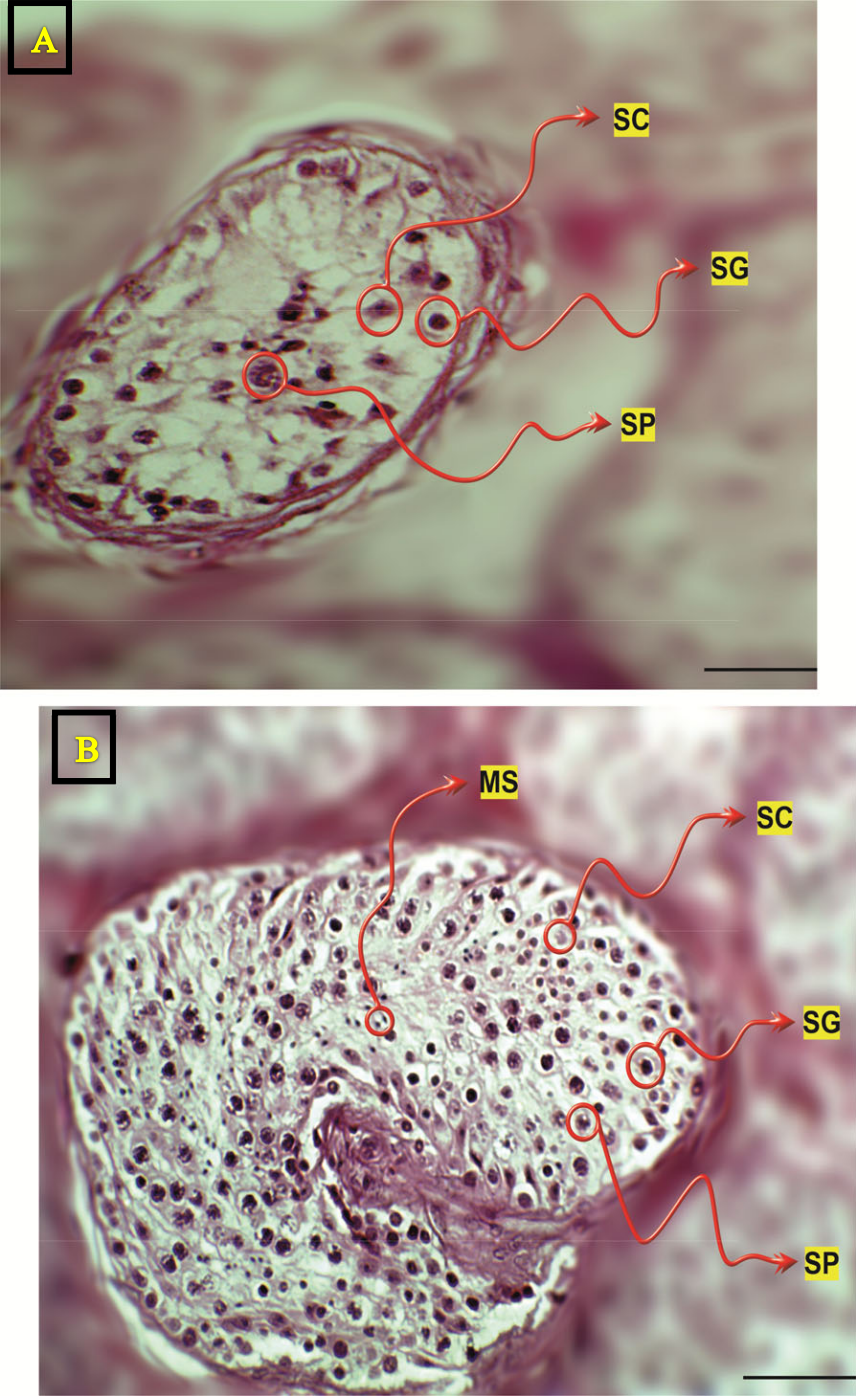
**a** Testicular segments of specimens with MA (maturation arrest). **b** Testicular segments of specimens with HP (hypospermatogenesis). SP = spermatocyte; MS = mature spermatid; SC = Sertoli cell; SG = spermatogonia; Scale bar = 20 μm

**Fig. 3:**
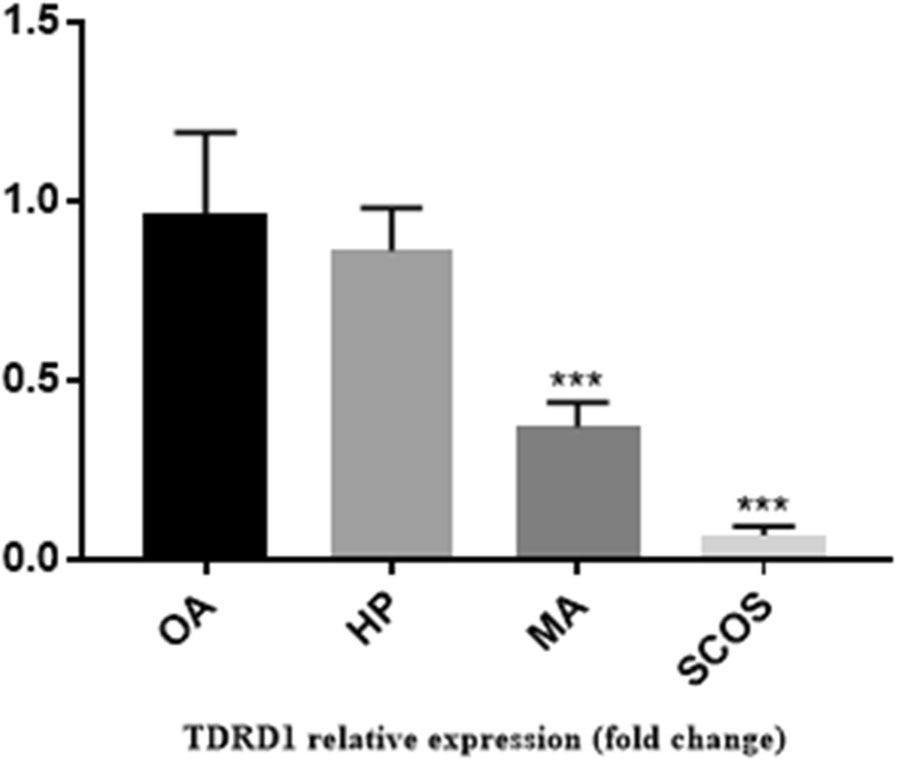
Comparison of the expression levels of *TDRD1* between HP, MA, SCOS, and OA(control) patients. *** *p* < 0.001

**Fig. 4:**
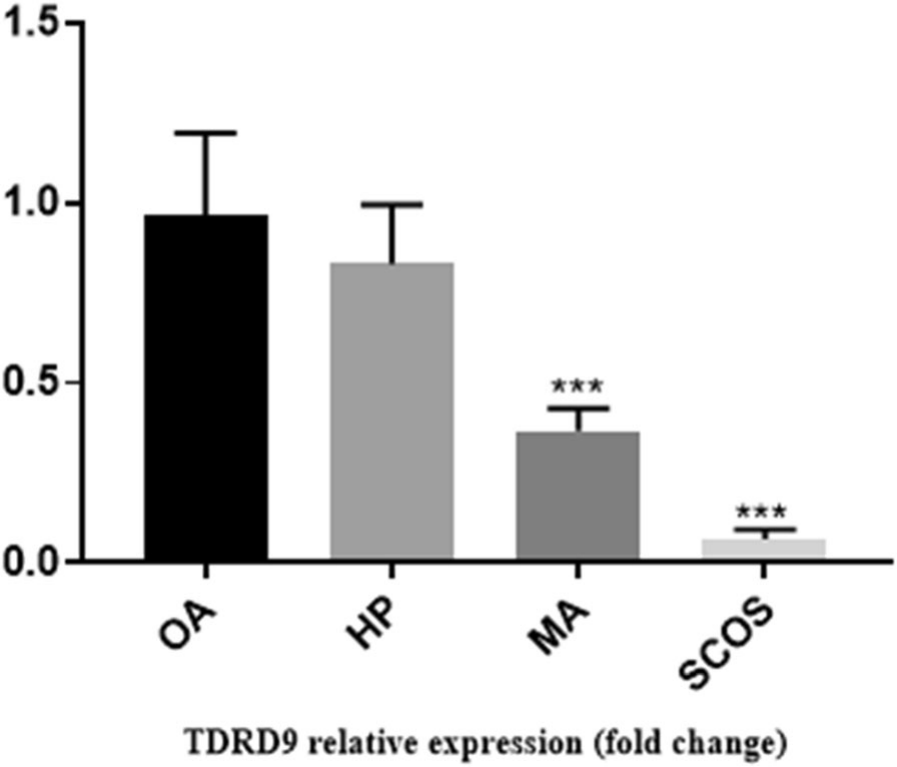
Comparison of the expression levels of TDRD9 between HP, MA, SCOS, and OA(control) patients. *** *p* < 0.001

**Fig. 5:**
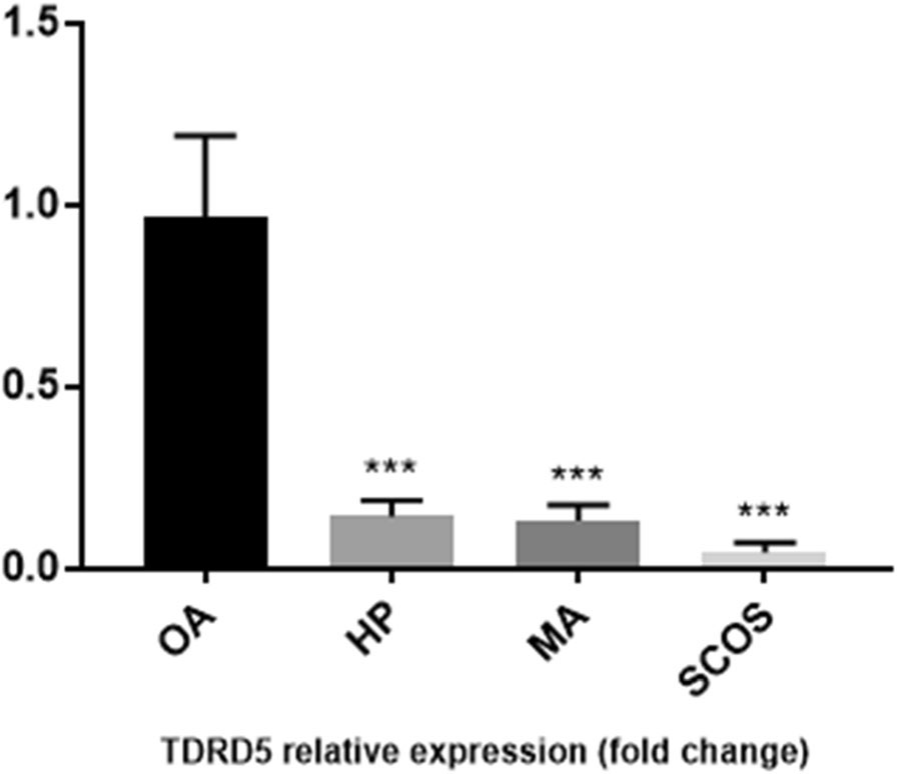
Comparison of the expression levels of *TDRD5* between HP, MA, SCOS, and OA(control) patients. *** *p* < 0.001

**Fig. 6:**
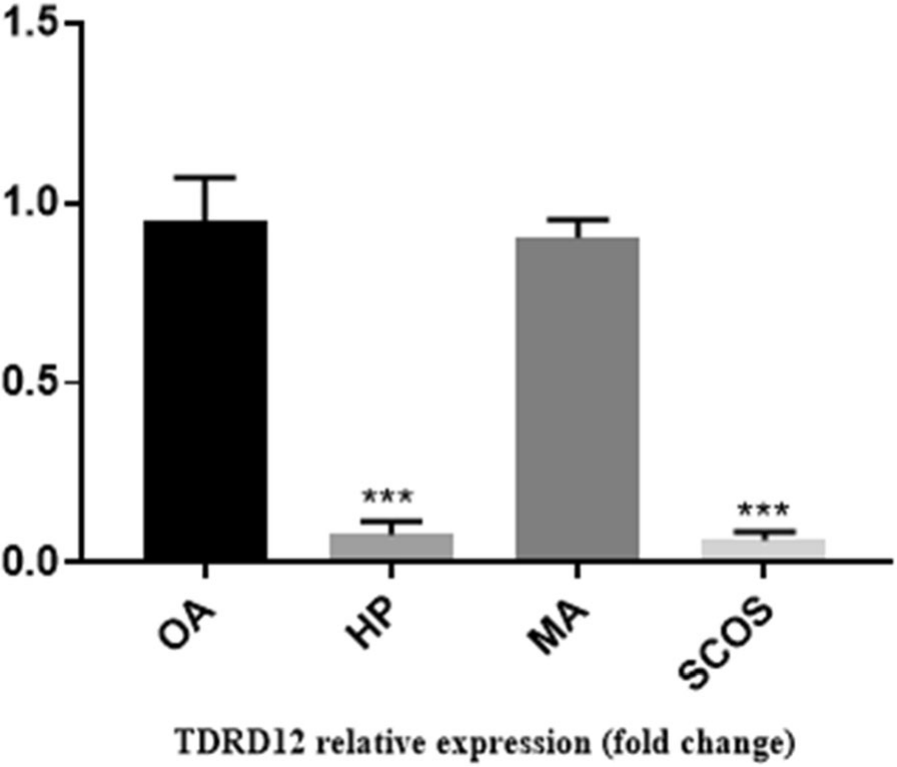
Comparison of the expression levels of TDRD12 between HP, MA, SCOS, and OA(control) patients. *** *p* < 0.001

**Fig. 7:**
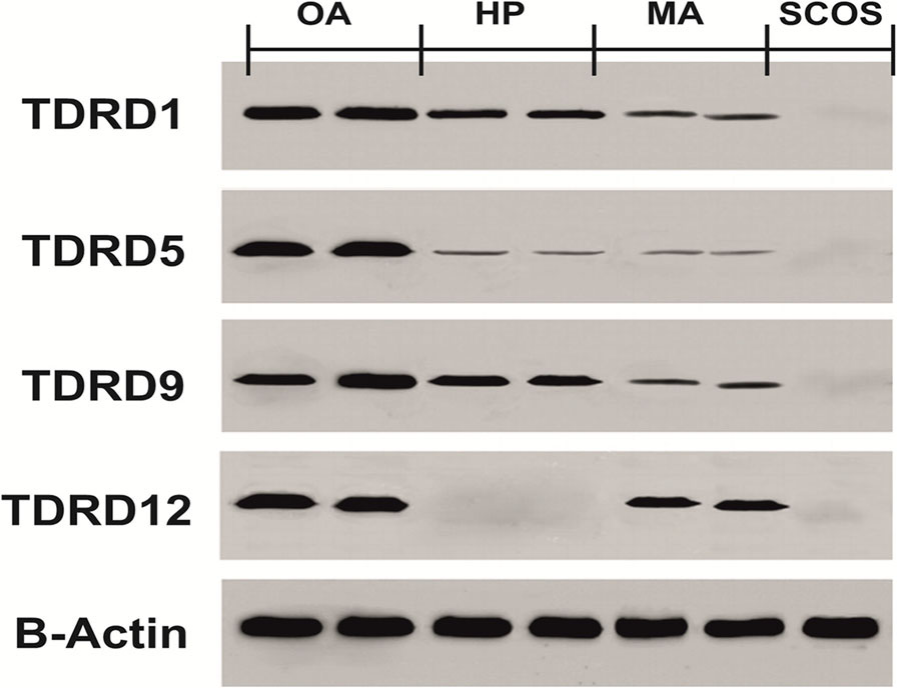
Western blotting test results for protein expression of *TDRDs* genes in HP, MA, SCOS, and OA samples compared to control group

Histological analysis of adult testis sections with hematoxylin and eosin (H&E) staining showed clear differences between samples (Figs. 1 and 2). Testicular *TDRD* gene expression was analyzed using qPCR and significant differences in expression observed between cases and controls. The expression of *TDRD1*, *TDRD5*, *TDRD9* and *TDRD12* genes were significantly lower in SCOS than controls (*P* < 0.001, Dunn’s post-test). The expression of *TDRD1* and *TDRD9* was significantly lower in MA samples than controls (*P* < 0.001, Dunn’s post-test) (Figs. 3 and 4). The expression of *TDRD5* was significantly lower in all cases compared to controls (P < 0.001, Dunn’s post-test) (Fig. 5). The results showed that the expression of *TDRD12* was also significantly lower in HP samples compared with controls (*P* < 0.001, Dunn’s post-test) (Fig. 6). The western blotting test showed the following results: TDRD1, TDRD5, TDRD9, and TDRD12 proteins were not expressed in SCOS samples. TDRD1 and TDRD9 proteins showed low expression in MA samples. The expression level of TDRD5 protein in MA and HP specimens was very low. TDRD12 protein was not expressed in HP samples (Fig. 7).

## Discussion

Spermatogenesis is a complex process, involving many cell type- and stage-specific transcription factors [20, 21]. Transposons have a major impact on the architecture and function of genomes, and thus their suppression is particularly important in early stage germ cells where epigenetic control is relaxed to permit genome-wide reprogramming. The large class of TDRDs play a pivotal role in the protection of germ cells against transposons [22, 23]; however, up to now their role in human spermatogenesis has not been well studied. The present study investigated the gene expression and protein levels of *TDRD1, TDRD5, TDRD9* and *TDRD12* genes in testicular tissue of NOA patients in comparison with OA patients.

Recent studies in mice models have demonstrated that Tdrd5 gene plays an important role in silencing of LINE-1, a transposon member of the long interspersed nuclear elements (LINE), which is involved in assembly of polar granules in germ cells, chromatoid bodies, and spermatogenesis [24, 25]. Previous projects showed that TDRD1 in prospermatogonia, arrange a complex with Mili (mouse homologue of PIWIL2), also TDRD9 is a vital partner of Miwi2 (mouse homologue of PIWIL4). The studies proved that the TDRD1 and TDRD9 are involved in biogenesis of primary and secondary piRNAs, transposon repression and accuracy of meiosis phase [15, 26–29]. TDRD12 has a crucial role in the final stage of meiosis phase, secondary piRNAs biogenesis and transposon silencing.

The lack of expression of *TDRD* genes found in SCOS was a common observation in this study, suggesting that the transcription of these genes could be limited to germ cells. Due to the lack of germ cells in SCOS, it seems likely that the expression levels of *TDRD* genes in the SCOS samples would not be detected. However, we did detect very low levels of expression of these genes in some individuals, which could show the existence of small foci of spermatogenesis in these specimens or mixed patterns in NOA subtypes. Western blot analysis results confirmed the findings from qPCR, and demonstrated lack of TDRD expression in SCOS samples. Because the expression of the *TDRDs* genes was found to be very limited in the qPCR technique in SCOS specimens, but not in the Western blot technique, this difference could be attributed to the inherent nature of the Western blot technique, which does not reveal very low protein expression.

We also found that *TDRD1* expression was significantly downregulated in the MA group versus controls. In *Mili* knockout mouse models, infertility of male mice has been shown to be due to stopping spermatogenesis at zygotene-pachytene stages. It has been revealed that hypermethylation of TDRD1 may lead to spermatogenic dysfunction [30]. Xiao-Bin Zhu et al. [31] identified the SNP rs77559927 in *TDRD1* was related to a diminished risk of spermatogenic malfunction [29]. Western blot analysis results confirmed that the protein encoded by the *TDRD1* gene had low expression in MA samples. The results of this experiment could confirm the role of TDRD1 in the early stages of meiosis process, likely to suppress transposons, which is essential for the progression of human spermatogenesis.

Another finding of this study was that the expression of *TDRD5* was significantly reduced in almost all cases (SCOS, MA and HP) compared to controls. Mitinori Saitou et al. reported that loss of *Tdrd5* leads to absence of spermatozoa, transposon depression and infertility [25]. *Tdrd5*^*−/−*^ mutations were arrested round spermatid and disorganized of chromatoid bodies [24]. The results of Western blot analysis also showed that the TDRD5 protein had a very low expression in almost all samples. These results revealed that TDRD5 is not restricted to prospermatogonia, unlike TDRD1, TDRD5 has more activity [25]. TDRD5 could have the same function in humans and mouse models, which plays a central role in the whole process of spermatogenesis.

The *TDRD9*, in collaboration with the *HIWI2* (human homologue of PIWIL4), has a substantial effect on the synthesis of the piRNAs. In *Tdrd9*^*−/−*^ mutants, the whole quantity of piRNAs were entirely reduced in testis [27]. Faruk Hadziselimovic et al. showed that cryptorchid boys with defected expression of *TDRD9* have a high risk of infertility due to transposon desilencing [32]. Two separate studies in the population of Iran and China reported a significant relationship between *HIWI2* gene rs508485 polymorphism and increased risk of spermatogenesis defects [33, 34]. Arafat et al. reported the relationship between the deletion frameshift mutation in *TDRD9* and NOA [35]. In present study, expression of *TDRD9* like *TDRD1* showed a significantly decreased in the MA samples compared with OA samples. Western blot analysis results proved that the protein encoded by the *TDRD9* gene had low expression in MA samples. It seems *TDRD9* and *TDRD1* have approximately the same role in human and other animals and they are very important for the meiosis process.

The TDRD12 is indirectly related to the TDRD1 and MILI, because it has a different location than they [36]. Jung-Jae Ko and colleagues revealed that TDRD12 is predominantly localized at the acrosome of the spermatid, which is completely different with other members of the TDRDs family [37]. In this study, *TDRD12* exhibited a very low expression in HP specimens in comparison to OA specimens. In addition, Western blot analysis results proved that the protein encoded by the *TDRD12* gene was not expressed in HP specimens. *TDRD12* is contributing to a process that leads to compaction of the sperm nucleus, histone-to-protamine exchange, which is essential for the differentiation of round spermatid into spermatozoa [36]. TDRD12 could be part of the master regulators in the terminal processes of spermatogenesis, and might act without piRNAs. piRNAs are inactivated in the round spermatid stage [38].

## Conclusion

To our knowledge, this study is the first to evaluate the expression of *TDRD1, TDRD5, TDRD9* and *TDRD12* genes in NOA and OA samples by qPCR and Western blot technique. Given the potential role for TDRDs in male infertility, our results suggest there is a need for further investigation to improve knowledge about the etiology of male infertility.

## Data Availability

The datasets generated and/or analyzed during the current study are available in the Google repository (following links). https://drive.google.com/uc?export=download&id=1V3IMH89ML0KK78Mhisa1BJLzsYKR84cB and https://drive.google.com/uc?export=download&id=10NJDXr7SO4ib0wlwLhfT2ds616w1seZF

## Abbreviations

HP: Hypospermatogenesis
LINE-1: Long interspersed nuclear elements
MA: Maturation arrest
Mili: Mouse homologue of PIWIL2
Miwi2: Mouse homologue of PIWIL4
NOA: Non-obstructive azoospermia
OA: Obstructive azoospermia
piRNA: Piwi-interacting RNA
PIWI: Piwi like RNA-Mediated Gene Silencing
SCOS: Sertoli cell-only syndrome
sDMA: Symmetric dimethylated arginine
TDRD: Tudor domain-containing proteins
